# Impacts of conventional and high-oleic soybean oil supplementation on rumen microbiology, physiology, immunity, and growth of recently weaned Brangus steers

**DOI:** 10.1093/tas/txag099

**Published:** 2026-07-16

**Authors:** Marcelo Vedovatto, Hiam Marcon, Matheus F L Ferreira, Ashley K Edwards, Joao V Chinaglia, Gilmara P Leite, Kelsey M Harvey, Brooklyn Laubinger, Barbara R dos Reis

**Affiliations:** Dean Lee Research and Extension Center, Louisiana State University, Alexandria, LA 71302, United States; Dean Lee Research and Extension Center, Louisiana State University, Alexandria, LA 71302, United States; Hill Farm Research Station, Louisiana State University, Homer, LA 71040, United States; Dean Lee Research and Extension Center, Louisiana State University, Alexandria, LA 71302, United States; Hill Farm Research Station, Louisiana State University, Homer, LA 71040, United States; Dean Lee Research and Extension Center, Louisiana State University, Alexandria, LA 71302, United States; Dean Lee Research and Extension Center, Louisiana State University, Alexandria, LA 71302, United States; Prairie Research Unit, Mississippi State University, Prairie, MS 39756, United States; Prairie Research Unit, Mississippi State University, Prairie, MS 39756, United States; CREC-White Sand Research Unit, Mississippi State University, Poplarville, MS 39470, United States

**Keywords:** cortisol, fatty acid profile, metabolites, preconditioning, vaccine response

## Abstract

The current experiment evaluated the effects of conventional (CSBO) and high-oleic soybean oils (HOSBO) on rumen microbiology, physiology, immunity, and growth of beef steers during a 56-day preconditioning period. Seventy-two Brangus steers (body weight [BW] = 248 ± 30.6 kg) were assigned to 1 of 3 treatments (4 pastures per treatment; 6 steers/pasture): (i) no oil (Control), (ii) CSBO, or (iii) HOSBO. Oils were supplemented at 0.06% BW/day mixed with soybean hulls, which were offered at 0.8% BW/day. Bermudagrass hay was provided ad libitum. Steers were vaccinated against respiratory diseases on day 0, and BW and blood samples were collected on days 0, 21, and 56, while rumen fluid was collected on days 0 and 56. The CSBO steers had lower rumen bacterial richness (*P* = 0.05) and a tendency toward lower Shannon indices (*P* = 0.10) than others at the phylum level. Furthermore, HOSBO steers had greater plasma oleic acid concentrations (*P* = 0.05) than other steers on days 21 and 56. The CSBO steers had greater plasma linoleic acid concentrations (*P* < 0.01) than HOSBO steers, which in turn had greater concentrations than Control steers on days 21 and 56. The HOSBO steers tended to have greater plasma α-linolenic acid concentrations (*P* = 0.08) than others on day 21. However, CSBO steers had greater plasma cortisol concentrations (*P* = 0.05) than Control steers and did not differ from HOSBO steers on day 21. In addition, CSBO steers tended to have greater serum alanine aminotransferase concentrations (*P* = 0.09) on day 56 than others. The CSBO steers had greater serum titers (*P* ≤ 0.05) against bovine viral diarrhea virus types 1 (BVDV-1) and 2 (BVDV-2) than others. Moreover, HOSBO steers had greater serum titers against bovine respiratory syncytial virus (BRSV; *P* = 0.03) on day 21 than others. Additionally, on day 56, HOSBO steers tended (*P* = 0.10) to have greater BW than CSBO steers, which in turn tended (*P* = 0.10) to have greater BW than Control steers. Thus, HOSBO and CSBO supplementation had a low effect on rumen microbiology but modified plasma fatty acid profiles, increasing linoleic acid with CSBO and oleic acid with HOSBO supplementation. These changes resulted in distinct immune responses, with CSBO enhancing responses to BVDV-1 and BVDV-2, and HOSBO enhancing responses to BRSV. Moreover, HOSBO was more effective than CSBO in supporting the growth of recently weaned Brangus steers.

## Teaser

Conventional and high-oleic soybean oils elicit distinct vaccine responses, and high-oleic oil supports greater growth than its conventional counterpart.

## Introduction

Conventional soybean oils (CSBO) have been used in ruminant nutrition as alternatives to increase the energy content of the diet or to provide specific fatty acids (FA; eg linoleic acid and ω-3 and ω-6 FA [NASEM 2016]). However, polyunsaturated FA (PUFA), when consumed by ruminants, are subjected to rumen biohydrogenation by bacteria that convert these high-quality FA into saturated FA (ie lower quality), which are then transported to the intestines for absorption (Jenkins 1993; Jenkins et al. 2008). Additionally, feeding high quantities of PUFA, or exceeding 6% of ether extract (EE) in the total diet consumed, is known to reduce the digestibility of non-lipid energy sources, especially fiber (Jenkins 1993; Jenkins et al. 2008; Jenkins and Harvatine 2014).

In contrast, high-oleic soybean oil (HOSBO) is rapidly gaining popularity due to its very high stability, reduced oxidation, and long shelf life (Knowlton 2022). Oleic acid, a monounsaturated FA (MUFA), is expected to undergo less biohydrogenation in the rumen, allowing a greater proportion of the original FA to be absorbed in the intestines (Jenkins 1993; Jenkins et al. 2008; Shirley 2019). Several experiments tested high-oleic soybean products in dairy cow diets, demonstrating their potential to increase milk fat content due to the greater oleic acid absorption (Lopes et al. 2017; Weld and Armentano 2018). However, only one experiment testing HOSBO in beef cattle diets was found in the literature, in which adding 3% HOSBO or CSBO to a grain-based diet did not affect intake or improve growth in confined finishing steers (Shirley 2019). However, it increased oleic acid concentrations in the abomasum, indicating greater availability of this FA for intestinal absorption. No plasma FA profile was evaluated in those steers, but it is expected to differ depending on the type of oil supplied (Shirley 2019). Despite substantial evidence that oleic acid can enhance immune function and overall health, most research to date has been conducted in laboratory animals and humans rather than in livestock, as observed in a recent published review (Santa-María et al. 2023). According to these authors, oleic acid exerts immunomodulatory effects primarily through its antioxidant and anti-inflammatory properties (Santa-María et al. 2023). Oleic acid is less susceptible to lipid peroxidation than PUFAs and therefore reduces oxidative stress in immune cells while suppressing proinflammatory cytokine production, thereby creating a more regulated immune environment (Yang et al. 2022; Santa-María et al. 2023). However, no studies have directly evaluated the effects of HOSBO supplementation on livestock immune systems, nor have they compared the immunological effects of CSBO vs HOSBO supplementation, underscoring the need for research in this area.

In beef cattle, weaning is a highly stressful event for calves, exposing them to numerous physiological challenges that directly impact their performance, health, and welfare (Galyean et al. 2022). Upon weaning, calves face multiple stressors, including maternal separation and changes in their social and physical environments. Additionally, weaning is often a time of various handling procedures, including vaccination, deworming, dehorning, castration, branding, and frequent commingling with new groups, which significantly impair the immune system (Lynch et al. 2019; Galyean et al. 2022). Consequently, a preconditioning period is typically implemented after weaning for grazing calves to facilitate adaptation and improve health before entering the subsequent confined phase (Galyean et al. 2022).

Based on the distinct ruminal and physiological characteristics of PUFA-rich CSBO and MUFA-rich HOSBO, we hypothesized that the type of soybean oil supplemented would differentially affect rumen microbiology composition, plasma FA profiles, and immune responses. We further expected that these differences would lead to improved vaccination responses and growth of steers supplemented with HOSBO compared with CSBO during the preconditioning period. We also hypothesized that supplementing soybean oil, independently of oil type, would improve the vaccination response and growth of steers. Thus, the objective of the current experiment was to evaluate the effects of CSBO and HOSBO supplementation on rumen microbiology, physiology, immunity, and growth of recently weaned Brangus steers kept in a forage-based system.

## Materials and methods

The experiment was conducted at Iberia Research Station (Jeanerette, LA; 29°57′32.52″ N 91°42′40.76″ W) of the Louisiana State University Agricultural Center (LSU AgCenter). All practices utilized were approved by the LSU AgCenter Animal Care and Use Committee (#A2024-12).

### Animals, treatments, and experimental design

Seventy-two recently weaned (14 days post-weaning) Brangus steers were stratified by body weight (BW; initial BW** = **248 ± 30.6 kg) and assigned to one of 12 pastures (1.35 ha each; 6 steers per pasture) on day 0 for a 56-day preconditioning period. Pastures were assigned to one of three treatments (4 pastures per treatment): (i) no oil supplementation (Control); (ii) CSBO, or (iii) HOSBO. Both oils were extracted and provided by the same company (Grain States Soya, West Point, NE) using a solvent‑free mechanical expeller process. Oils were supplemented at 0.06% of BW/day (dry matter [DM] basis) and top-dressed onto soybean hulls. This oil inclusion rate was selected to ensure that the EE concentration in the total diet consumed was close but not exceeding 6%, thereby avoiding potential adverse effects on ruminal fermentation (NASEM 2016). Soybean hulls were offered at 0.8% of BW/day (DM basis), whereas the Control groups received only soybean hulls without added oil. Oil and soybean hull quantities were adjusted based on BW on days 0 and 21. The quantity of oil and soybean hulls required per pasture/week was calculated, then divided by 3, and fed three times per week (Monday, Wednesday, and Friday) at 0800 hours, and was fully consumed by all steers within approximately 45 minutes.

All pastures were mowed and fully grazed before the start of the experiment. Because the experiment was conducted during the fall season, no measurable forage regrowth occurred during the experimental period; therefore, bermudagrass hay (*Cynodon dactylon*) was offered ad libitum in round bales placed in hay rings, replacing the bales as needed. During the entire experiment, animals also had free-choice access to mineral supplement (guaranteed levels of 15–18% Ca, 5% P, 11–13.2% Salt, 3% Mg, 0.10% K, 25 mg/kg Co, 2000 mg/kg Cu, 150 mg/kg I, 26.4 mg/kg Se, 6000 mg/kg Zn, 441 IU/kg vitamin A, 45 IU/kg vitamin D_3_ and 441 IU/kg vitamin E; mineral target intake of 113 g/head/d; 1068—Breeder Mineral, Lone Star Feed, Nacogdoches, TX).

Twenty-eight days before weaning, and again on day 0, steers were vaccinated against respiratory diseases (bovine rhinotracheitis [IBR], parainfluenza-3 virus [PI_3_], bovine viral diarrhea virus type 1 and 2 [BVDV-1 and BVDV-2], bovine respiratory syncytial virus [BRSV], and *Mannheimia haemolytica* [2 mL s.c.; Bovi Shield Gold One Shot, Zoetis, Parsippany, NJ]). Additionally, twenty-eight days before weaning, steers were vaccinated against clostridial diseases (2 mL subcutaneously; Ultrabac 7, Zoetis) and administered an oral anthelmintic (5 mg/kg of BW; Safe-guard, Merck Animal Health, Summit, NJ) to protect against internal parasites.

### Data and sample collection

Hay, soybean hulls, and oil samples were collected weekly, and a composite sample was prepared for subsequent chemical and FA concentration analyses. Feed samples (except oil) were dried in a forced-air oven at 55°C for 72 h before being submitted to laboratory analysis.

Body weight was collected from all steers, and blood samples were collected from a subset of 36 steers (12 steers/treatment; 3 steers/pasture) on days 0, 21, and 56. Collections on days 21 and 56 were performed 24 hours after the feeding of oils and soybean hulls. Blood samples were collected from the jugular vein into (i) 10-mL sodium-heparin-containing tubes (Vacutainer, Becton Dickson, Franklin Lakes, NJ) to determine plasma concentrations of insulin-like growth factor 1 (IGF-1), cortisol, insulin and FA profile; and (ii) into 10-mL tubes containing no additive (Vacutainer, Becton Dickson) to evaluate the concentration of serum antibody titers against IBR, PI_3_, BVDV-1 and 2 and BRSV viruses and serum biochemistry profile. Immediately following collection, blood samples were placed on ice and then centrifuged at 2000 × *g* for 20 minutes at 4°C. Serum and plasma samples were stored at −80°C until further laboratory analysis.

Rumen fluid was collected from a subset of 36 steers (12 steers/treatment; 3 steers/pasture) on days 0 and 56 (24 hours after the previous oil and soybean hulls feeding) using oral stomach tubing and a vacuum pump. The first 100 mL of rumen fluid was discarded to prevent contamination with saliva. The following 300 mL of rumen fluid was filtered through four layers of cheesecloth. Then, a subsample was transferred into pre-labeled 15 mL centrifuge tubes for further microbiological analysis. Immediately after collection, samples were placed on dry ice and later stored at −80°C until further analysis (De Mulder et al. 2018).

### Laboratory analysis

Feed samples were sent to a commercial laboratory (Dairy One Forage Laboratory, Ithaca, NY) and analyzed by wet procedures for crude protein (CP), neutral detergent fiber (NDF), acid detergent fiber (ADF), and ether extract (Table 1). Total digestible nutrients (TDN) concentrations were calculated as described by Weiss et al. (1992), while net energy for maintenance (NE_m_) and gain (NE_g_) were estimated using the equations proposed by NASEM (2016). Feed and plasma FA compositions were analyzed in our laboratory using gas chromatographic equipment (Agilent 7890, Agilent Technologies, Inc., Santa Clara, CA) according to Tripathy et al. (2010) and presented in Table 2.

**Table 1:** Chemical composition [dry matter (DM) basis] of hay and soybean hulls offered to steers during the 56-day preconditioning period^1^.

Item^2^	Bermudagrass hay	Soybean hulls
**CP, % DM**	6.50	13.0
**NDF % DM**	73.1	60.3
**ADF % DM**	45.1	47.7
**EE, % DM**	1.96	2.71
**TDN^3^, % DM**	54.0	63.0
**NEm^4^, Mcal/kg DM**	0.99	1.32
**NEg^4^, Mcal/kg DM**	0.44	0.74

1 Hay was offered ad libitum, and soybean hulls were fed at 0.8% of body weight (BW). The Control group received no oil, whereas the conventional soybean oil (CSBO) and high-oleic soybean oil (HOSBO) groups had oil top-dressed onto soybean hulls at a rate of 0.06% of BW per steer per day.

2 CP, crude protein; NDF, neutral detergent fiber; ADF, acid detergent fiber; TDN, total digestible nutrients; NEm, net energy for maintenance; NEg, net energy for growth. Samples were analyzed by wet procedures in a commercial laboratory (Dairy One Forage Laboratory, Ithaca, NY).

3 Calculated as described by Weiss et al. (1992).

4 Calculated using the equations proposed by the NASEM (2016). The TDN was first converted to digestible energy (DE) using the formula DE (Mcal/kg) = 0.04409 × TDN. Digestible energy was then converted to metabolizable energy (ME) using ME (Mcal/kg) = 0.82 × DE. Finally, ME was converted to net energy values using the following equations: NEm (Mcal/kg) = 1.37 × ME − 0.138 × ME^2^ + 0.0105 × ME³ − 1.12 and NEg (Mcal/kg) = 1.42 × ME − 0.174 × ME^2^ + 0.0122 × ME³ − 1.65.

**Table 2:** Fatty acid (FA) profile (% of total FA detected) of hay, soybean hulls, conventional soybean oil (CSBO), and high-oleic soybean oil (HOSBO) offered to steers during the 56-day preconditioning period^1^.

Item^2^	Bermudagrass hay	Soybean hulls	CSBO	HOSBO
**Myristic acid (14:0)**	0.71	ND^3^	ND^3^	ND^3^
**Pentadecenoic acid (15:1)**	0.31	ND^3^	ND^3^	ND^3^
**Palmitic acid (16:0)**	34.6	14.1	10.7	7.79
**Palmitoleic acid (16:1, ω-7)**	1.50	ND^3^	ND^3^	ND^3^
**Heptadecanoic acid (17:0)**	1.15	ND^3^	ND^3^	ND^3^
**Stearic acid (18:0)**	6.75	5.37	5.71	4.77
**Oleic acid (18:1, ω-9)**	13.8	22.9	30.1	69.5
**Linoleic acid (18:2, ω-6)**	21.6	47.2	46.3	14.2
**α-Linolenic acid (18:3, ω-3)**	16.4	10.1	6.81	2.92
**Arachidic acid (20:0)**	1.38	ND^3^	0.31	ND^3^
**Eicosapentaenoic acid (20:5, ω-3)**	1.34	0.33	ND^3^	ND^3^
**Docosadienoic acid (22:2, ω-6)**	0.43	ND^3^	ND^3^	ND^3^
**Total SFA**	44.6	19.5	16.7	13.3
**Total MUFA**	15.6	22.9	30.1	69.5
**Total ω-6 PUFA**	22.0	47.2	46.3	14.2
**Total ω-3 PUFA**	17.7	10.4	6.81	2.92
**ω-6/ω-3 PUFA ratio**	1.24	4.54	6.80	4.87

1 Hay was offered ad libitum, and soybean hulls were fed at 0.8% of body weight (BW). The Control group received no oil, whereas the CSBO and HOSBO groups had oil top-dressed onto soybean hulls at a rate of 0.06% of BW per steer per day.

2 SFA, saturated fatty acids; MUFA, monounsaturated fatty acids; PUFA, polyunsaturated fatty acids. The FA were analyzed using a gas chromatograph equipment (Agilent 7890, Agilent Technologies, Inc., Santa Clara, CA) according to Tripathy et al. (2010).

3 Not detected.

Rumen fluid samples were used for microbial analysis, in which genomic DNA was extracted and submitted to PathoSense BV (Lier and Ghent, Belgium) for 16S rRNA gene sequencing following the protocols described by De Mulder et al. (2018). Alpha diversity indices (richness, Shannon index, and Simpson index) were calculated to characterize within-sample bacterial diversity. Bacterial richness was defined as the total number of distinct bacterial taxa detected per sample, reflecting the number of different genera and phyla present. The Shannon index was calculated to describe overall bacterial diversity by jointly accounting for both the number of taxa and their relative distribution (evenness) within each sample (Shannon 1948). The Simpson index was calculated to assess community dominance, giving greater weight to the most abundant taxa within each sample (Simpson 1949).

Serum concentrations of IGF-1, insulin, and cortisol were measured using chemiluminescent enzyme immunoassays (Immulite 2000; Siemens Medical Solutions Diagnostics, Los Angeles, CA), with intra- and inter-assay coefficients of variation < 10%. Serum biochemistry analyses were performed using a Beckman Coulter AU480 Chemistry Analyzer (Beckman Coulter Inc., Brea, CA). Concentrations of albumin, total protein, urea nitrogen, glucose, and creatinine, as well as the enzymatic activities of alanine aminotransferase (ALT), aspartate aminotransferase (AST), and gamma-glutamyl transferase (GGT), were measured using colorimetric and enzymatic kits from Beckman Coulter Inc., with intra- and inter-assay coefficients of variation < 10%. Globulin concentrations were calculated by subtracting albumin from total protein.

Antibody titers against IBR, PI_3_, BVDV-1, BVDV-2, and BRSV were analyzed at the Oklahoma Animal Disease Diagnostic Laboratory (Stillwater, OK) following the procedures described by Rosenbaum et al. (1970). Individual serum samples were evaluated for the greatest dilution that achieved complete cell protection against each virus, and results are reported as log_2_ values. Serum neutralization titers < 4 were considered negative and assigned a value of 0, whereas titers ≥ 4 were considered positive and assigned a value of 1. Seroconversion for each virus was calculated as the percentage of steers that exhibited a positive serum neutralization titer response (Richeson et al. 2008).

### Statistical analysis

The experiment used a completely randomized design, with pasture as the experimental unit for all statistical analyses. Sample size calculations were performed prior to the execution of the experiment using the POWER procedure in SAS (version 9.4; SAS Institute Inc., Cary, NC, USA). The number of pastures (*n* = 4/treatment) and steers (24 for BW and 12/treatment for all other variables) was sufficient to detect at least 10% difference between treatments for the primary outcome variables, assuming *P* = 0.05 and 80% power based on a two-sided *t*-test. Sample size estimates were derived from a previous experiment conducted by our group in the same location site (Vedovatto et al. 2024).

Data collected were analyzed using the GLIMMIX (for binary data; only seroconversion data) and MIXED (for non-binary data; all other data) procedures in SAS. The Satterthwaite approximation was used to determine the denominator degrees of freedom for the fixed-effects test. The statistical model for average daily gain (ADG) included fixed effects for treatment and random effects for pasture (treatment) and animal (pasture). All other variables were analyzed as repeated measures, and the statistical model included fixed effects for treatment, day, and the treatment-by-day interaction, as well as random effects for pasture (treatment) and animal (pasture). The day 0 values for each variable were included as covariates but removed from the model if *P* > 0.10. The covariance structures tested were the autoregressive-1, compound-symmetry, unstructured, and Toeplitz. The autoregressive 1 structure was selected for all data because it yielded the lowest Akaike information criterion, indicating the best fit for the repeated-measures data. Means were separated using the protected least significant difference (*t*-test), and all results were reported as least square means followed by SEM. Significance was defined as *P* ≤ 0.05, and tendency as *P* > 0.05 and ≤ 0.10.

## Results

Results are reported according to the highest-order interaction detected. Treatments did not affect (*P* ≥ 0.17) rumen bacterial phylum-level composition, phylum-level alpha diversity, or genus-level bacterial composition (Table 3). However, at the genus level, CSBO steers exhibited lower rumen bacterial richness (*P* = 0.05) and tended to have lower Shannon diversity indices (*P* = 0.10) than steers receiving other treatments. No effects of treatments were detected (*P* = 0.57) for the genus-level Simpson index.

**Table 3:** Phylum- and genus-level bacterial composition and alpha diversity of rumen fluid of beef steers supplemented with no-oil (Control; *n* = 12), conventional soybean oil (CSBO; *n* = 12), or high-oleic soybean oil (HOSBO; *n* = 12) during the 56-day preconditioning period^1^.

Item	Treatments			SEM	*P*-value
	Control	CSBO	HOSBO		Treatment
***Phylum level***					
**Composition (relative abundance, %)**					
**Bacteroidetes^2^**	16.8	17.6	16.6	1.66	0.91
**Firmicutes**	77.5	76.9	78.4	1.95	0.87
**Proteobacteria**	2.56	2.82	3.03	0.24	0.38
**Spirochaetes^2^**	1.83	1.93	1.69	0.20	0.72
**Candidatus Melainabacteria**	2.66	3.89	3.17	0.56	0.32
**Tenericutes**	1.49	1.39	1.49	0.25	0.94
**Alpha-diversity**					
**Richness**	19.2	18.9	19.3	0.48	0.84
**Shannon index**	0.76	0.76	0.73	0.04	0.84
**Simpson Index**	0.36	0.37	0.35	0.02	0.82
***Genus level***					
**Composition (relative abundance, %)**					
***Prevotella***	10.8	12.2	10.2	1.51	0.61
***Ruminococcus***	14.3	15.6	14.01	1.18	0.64
***Eubacterium***	8.03	7.92	7.97	0.60	0.99
***Christensenella***	12.1	10.5	12.1	0.82	0.32
***Butyrivibrio***	4.61	5.27	5.20	0.32	0.33
***Lachnoclostridium***	4.18	4.97	4.89	0.39	0.32
***Saccharofermentans*^2^**	3.56	2.67	2.64	0.62	0.55
***Roseburia***	1.50	1.87	1.42	0.21	0.37
***Succiniclasticum***	1.93	1.87	1.87	0.15	0.95
***Treponemas*^2^**	1.80	1.91	1.66	0.20	0.69
***Selenomonas*^2^**	1.23	1.42	1.51	0.21	0.66
***Enterocloster***	2.24	2.60	2.54	0.19	0.41
***Negativibacillus***	1.48	1.89	1.14	0.40	0.45
***Blautia***	0.95	1.18	0.98	0.09	0.17
***Anaerobutyricum***	1.49	1.44	1.39	0.12	0.85
***Anaeroplasma***	1.24	1.18	1.23	0.22	0.98
***Pseudobutyrivibrio***	1.75	0.93	1.15	0.18	0.60
**Alpha-diversity**					
**Richness**	188^a^	174^b^	191^a^	5.37	0.05
**Shannon index**	3.41^a^	3.35^b^	3.45^a^	0.03	0.10
**Simpson Index^2^**	0.93	0.92	0.93	0.003	0.57

1 Hay was offered ad libitum, and soybean hulls were fed at 0.8% of body weight (BW). The Control group received no oil, whereas the CSBO and HOSBO groups had oil top-dressed onto soybean hulls at a rate of 0.06% of BW per steer per day. Rumen samples were collected on days 0 and 56, and the results reported are for day 56. Only phyla and genera with relative average abundance ≥ 1% are presented.

2 Day 0 values were included as a covariate in the statistical model (*P* ≤ 0.09).

The CSBO and HOSBO supplementation affected the plasma concentration of several FAs (Table 4). The CSBO and HOSBO steers had greater (*P* = 0.04) palmitic acid concentrations than the Control steers. A treatment × day effect was detected (*P* = 0.05) for palmitoleic acid concentrations, which were greater for HOSBO steers compared to the others on day 21. Interestingly, a treatment × day effect was detected (*P* < 0.01) for stearic acid concentrations, which was greater for HOSBO vs. CSBO steers, which, in turn, had greater concentrations than Control steers on day 21; however, on day 56, CSBO steers had greater concentrations than the steers receiving other treatments. As expected, a treatment × day effect was detected (*P* = 0.05) for oleic acid concentrations, with HOSBO steers having a greater concentration than the others on days 21 and 56. A treatment × day effect was detected (*P* < 0.01) for linoleic acid concentrations, which CSBO steers had a greater concentration than HOSBO steers, which, in turn, had greater concentrations than Control steers on days 21 and 56. Furthermore, a treatment × day effect tended to be detected (*P* = 0.08) for α-linolenic acid concentrations, with HOSBO steers tending to have greater concentrations than others on day 21. A treatment × day effect was detected (*P* < 0.01) for total FA concentrations, which HOSBO steers had greater concentrations than CSBO steers, which, in turn, had greater concentrations than Control steers on day 21; however, on day 56, CSBO steers had greater concentrations than HOSBO steers, which, in turn, had greater concentrations than Control steers. In addition, a treatment × day effect was detected (*P* = 0.01) for total saturated FAs (SFA) concentrations, with HOSBO steers having greater concentrations than others on day 21; however, on day 56, CSBO steers had greater concentrations than the others. A treatment × day effect tended to be detected (*P* = 0.07) for total MUFA concentrations, with HOSBO steers tending to have greater concentrations than others on days 21 and 56. A treatment × day effect was also detected (*P* < 0.01) for total PUFA ω-6 concentrations, which CSBO and HOSBO steers had greater concentrations than the Control steers on day 21; however, on day 56, CSBO steers had greater concentrations than HOSBO steers, which, in turn, had greater concentrations than the Control steers. A treatment × day effect was detected (*P* < 0.01) for total PUFA ω-3 concentrations, with HOSBO steers having greater concentrations than others on day 21. In addition, a treatment × day effect was detected for the ω-6/ω-3 PUFA ratio (*P* < 0.01), with CSBO steers having a greater ratio than others on day 21; however, on day 56, CSBO steers had a greater ratio than HOSBO steers, which in turn was greater than for Control steers. No other effects of treatment and treatment × day were detected (*P* ≥ 0.17) for any other FA concentration.

**Table 4:** Plasma fatty acid concentration (ug/mL) of beef steers supplemented with no-oil (Control; *n* = 12), conventional soybean oil (CSBO; *n* = 12), or high-oleic soybean oil (HOSBO; *n* = 12) during the 56-day preconditioning period^1^.

Item^2^^,^^3^	Treatments			SEM	*P*-value	
	Control	CSBO	HOSBO		Treatment	Treatment × day
**Pentadecanoic acid (15:0)**	6.89	9.41	5.96	7.84	0.93	0.34
**Palmitic acid (16:0)**	305^b^	355^a^	351^a^	15.4	0.04	0.11
**Palmitoleic acid (16:1, ω−7)**					0.23	0.05
**d 0**	32.3	29.0	27.6	3.30		
**d 21**	9.42^b^	6.51^b^	21.0^a^	3.45		
**d 56**	20.2	15.0	14.4	3.45		
**Heptadecanoic acid (17:0)**	25.3	29.7	34.6	4.20	0.31	0.17
**Stearic acid (18:0)**					0.02	<0.01
**d 0**	355	351	355	40.9		
**d 21**	338^c^	420^b^	584^a^	42.7		
**d 56**	419^b^	562^a^	459^b^	42.7		
**Oleic acid (18:1, ω−9)**					0.14	0.05
**d 0**	234	236	182	41.4		
**d 21**	184^b^	181^b^	340^a^	43.2		
**d 56**	65.0^b^	96^b^	175^a^	43.2		
**Linoleic acid (18:2, ω−6)**					<0.01	<0.01
**d 0**	408	397	392	48.9		
**d 21**	395^c^	778^a^	655^b^	51.1		
**d 56**	495^c^	959^a^	642^b^	51.1		
**γ-Linolenic acid (18:3, ω−6)**	29.7	25.1	35.6	5.25	0.38	0.20
**α-Linolenic acid (18:3, ω−3)**					0.30	0.08
**d 0**	120	109	113	9.48		
**d 21**	101^b^	115^b^	141^a^	9.90		
**d 56**	117	134	120	9.89		
**Arachidic acid (20:0)**	0.13	0.13	0.60	0.31	0.48	0.71
**Dihomo-γ-linolenic acid (20:3, ω−6)**	5.84	8.94	107	1.89	0.20	0.18
**Arachidonic acid (20:4, ω−6)**	17.8	22.5	20.1	4.72	0.76	0.28
**Eicosapentaenoic acid (20:5, ω−3)**	8.23	8.36	6.43	2.05	0.76	0.22
**Docosadienoic acid (22:2, ω−6)**	0.36	0.29	0.29	0.38	0.99	0.21
**Osbond acid (22:5, ω−6)**	4.14	6.04	3.62	2.62	0.79	0.78
**Docosapentaenoic acid (22:5, ω−3)**	4.80	4.17	8.08	3.41	0.39	0.14
**Docosahexaenoic acid (22:6, ω−3)**	0.26	0.26	0.34	0.32	0.98	0.23
**Lignoceric acid (24:0)**	0.70	1.31	0.70	0.57	0.68	0.89
**Total FA**					<0.01	<0.01
**d 0**	1636	1596	1576	133		
**d 21**	1347^c^	1853^b^	2230^a^	139		
**d 56**	1542^c^	2336^a^	1873^b^	139		
**Total SFA**					0.04	0.01
**d 0**	727	717	736	69.9		
**d 21**	627^b^	751^b^	987^a^	73.0		
**d 56**	780^b^	1038^a^	836^b^	73.0		
**Total MUFA**					0.13	0.07
**d 0**	270	267	227	42.2		
**d 21**	197^b^	191^b^	356^a^	44.0		
**d 56**	88.6^b^	114^b^	183^a^	44.0		
**Total PUFA ω−6**					<0.01	<0.01
**d 0**	490	482	469	51.6		
**d 21**	426^b^	797^a^	722^a^	53.9		
**d 56**	547^c^	1038^a^	723^b^	53.9		
**Total PUFA ω−3**					0.16	<0.01
**d 0**	149	133	137	10.7		
**d 21**	98.7^b^	118^b^	159^a^	11.2		
**d 56**	123	151	126	11.2		
**ω−6/ω−3 PUFA ratio**					<0.01	<0.01
**d 0**	3.41	3.87	3.53	0.35		
**d 21**	4.26^b^	6.88^a^	4.61^b^	0.36		
**d 56**	4.59^c^	7.28^a^	6.04^b^	0.36		

1 Hay was offered ad libitum, and soybean hulls were fed at 0.8% of body weight (BW). The Control group received no oil, whereas the CSBO and HOSBO groups had oil top-dressed onto soybean hulls at a rate of 0.06% of BW per steer per day.

2 FA, fatty acids; SFA, saturated fatty acids; MUFA, monounsaturated fatty acids; PUFA, polyunsaturated fatty acids. The FA were analyzed using a gas chromatograph equipment (Agilent 7890, Agilent Technologies, Inc., Santa Clara, CA) according to Tripathy et al. (2010).

3 Day 0 values were included as a covariate in the statistical model (*P* ≤ 0.03).

a,b Within a row, without a common superscript differ (*P* ≤ 0.05) or tend to differ (*P* ≤ 0.10).

No effects of treatment or treatment × day interactions were detected (*P* ≥ 0.16) for plasma concentrations of IGF-1 and insulin (Table 5). However, a treatment × day effect was detected (*P* = 0.05) for plasma cortisol concentrations, which CSBO steers had greater concentrations than Control steers and did not differ from HOSBO steers on day 21. In addition, treatment day effect tended to be detected (*P* = 0.09) for serum AST concentrations, which CSBO steers tended to have greater concentration than others on day 56. No other effects of treatment or treatment × day interactions were detected (*P* ≥ 0.23) for any other serum biochemistry variable.

**Table 5:** Plasma hormones and serum biochemistry of beef steers supplemented with no-oil (Control; *n* = 12), conventional soybean oil (CSBO; *n* = 12), or high-oleic soybean oil (HOSBO; *n* = 12) during the 56-day preconditioning period^1^.

Item^2^	Treatments			SEM	Reference values^3^	*P*-value	
	Control	CSBO	HOSBO			Treatment	Treatment × day
***Plasma hormones***							
**IGF-1, ng/mL^4^**	124	123	117	5.20		0.62	0.16
**Insulin, µIU/mL**	3.02	3.80	4.24	0.55	0–5	0.27	0.80
**Cortisol, ug/dL^4^**					0.61 ± 0.07	0.60	0.05
**d 0**	2.39	2.25	2.16	0.27			
**d 21**	1.49^b^	2.49^a^	2.14^ab^	0.27			
**d 56**	3.56	3.56	3.44	0.27			
***Serum biochemistry***							
**Albumin, g/dL^4^**	3.24	3.24	3.23	0.04	3.03–3.55	0.98	0.85
**Globulin, g/dL^4^**	3.48	3.40	3.42	0.11	3.00–3.48	0.63	0.91
**Total protein, g/dL^4^**	6.71	6.63	6.66	0.08	6.74–7.46	0.77	0.94
**Creatinine, mg/dL^4^**	1.60	1.62	1.64	0.03	1.00–2.00	0.72	0.32
**SUN, mg/dL^4^**	4.86	4.68	4.54	0.22	8–11	0.61	0.56
**Glucose, mg/dL^4^**	94.7	99.2	100	5.31	45–75	0.76	0.42
**AST, U/L^4^**					78–132	0.29	0.09
**d 0**	63.7	63.3	62.3	4.12			
**d 21**	78.0	83.6	81.5	4.30			
**d 56**	103^b^	113^a^	97.4^b^	4.51			
**ALT, U/L^4^**	26.4	24.0	24.2	0.98	11–40	0.23	0.37
**GGT, U/L**	17.2	13.6	15.0	3.40	6.1–17.4	0.52	0.64

1 Hay was offered ad libitum, and soybean hulls were fed at 0.8% of body weight (BW). The Control group received no oil, whereas the CSBO and HOSBO groups had oil top-dressed onto soybean hulls at a rate of 0.06% of BW per steer per day.

2 IGF-1, insulin-like growth factor 1; SUN, serum urea nitrogen; AST, aspartate aminotransferase; ALT, alanine aminotransferase; GGT, gamma-glutamyl transferase.

3 Normal physiological ranges according to Kaneko (2008).

4 Day 0 values were included as a covariate in the statistical model (*P* ≤ 0.02).

a,b Within a row, without a common superscript differ (*P* ≤ 0.05) or tend to differ (*P* ≤ 0.10).

Effects of treatments were detected (*P* ≤ 0.05) for response to vaccination, and CSBO steers had greater serum titers against BVDV-1 and BVDV-2 than steers receiving the other treatments (Table 6). In addition, a treatment × day effect was detected (*P* = 0.03) for serum titers against BRSV, with HOSBO steers having greater titers than others on day 21. Furthermore, a treatment × day effect was detected (*P* = 0.10) for BRSV seroconversion, with HOSBO steers showing greater seroconversion than others on day 21. No other effects of treatment or treatment × day interactions were detected (*P* ≥ 0.46) for any other response-to-vaccination variable.

**Table 6:** Response to respiratory vaccination of beef steers supplemented with no-oil (Control; *n* = 12), conventional soybean oil (CSBO; *n* = 12), or high-oleic soybean oil (HOSBO; *n* = 12) during the 56-day preconditioning period^1^.

Item^2^	Treatments			SEM	*P*-value	
	Control	CSBO	HOSBO		Treatment	Treatment × day
***Titer concentration, log_2_***						
**IBR^3^**	3.70	3.87	3.99	0.25	0.70	0.48
**PI_3_^3^**	6.31	6.37	6.30	0.42	0.99	0.99
**BVDV-1^3^**	7.60^b^	8.63^a^	7.80^b^	0.31	0.05	0.85
**BVDV-2^3^**	7.52^b^	8.08^a^	7.23^b^	0.23	0.04	0.15
**BRSV**					0.05	0.03
**d 0**	2.13	2.05	2.09	0.10		
**d 21**	2.04^b^	2.25^b^	2.64^a^	0.11		
**d 56**	2.13	2.14	2.27	0.11		
***Seroconversion, %***						
**IBR^3^**	68.7	67.3	71.6	6.35	0.89	0.55
**PI_3_^3^**	86.6	82.3	88.8	5.16	0.67	0.73
**BVDV-1^3^**	94.9	92.7	94.4	3.35	0.88	0.46
**BVDV-2**	100	100	100	–	–	–
**BRSV^3^**					0.18	0.10
**d 0**	7.10	4.24	7.09	9.21		
**d 21**	7.10^b^	2.40^b^	51.0^a^	9.99		
**d 56**	16.2	13.3	25.3	9.58		

1 Hay was offered ad libitum, and soybean hulls were fed at 0.8% of body weight (BW). The Control group received no oil, whereas the CSBO and HOSBO groups had oil top-dressed onto soybean hulls at a rate of 0.06% of BW per steer per day.

2 IBR, infectious bovine rhinotracheitis; BVDV-1, bovine viral diarrhea virus type 1; BVDV-2, bovine viral diarrhea virus type 2; BRSV, bovine respiratory syncytial virus. A vaccine against these viruses (2 mL subcutaneous; Bovi Shield Gold One Shot; Zoetis, Parsippany, NJ) was applied on day 0.

3 Day 0 values were included as a covariate in the statistical model (*P* ≤ 0.01).

a,b Within a row, without a common superscript differ (*P* ≤ 0.05) or tend to differ (*P* ≤ 0.10).

A tendency for a treatment × day interaction (*P* = 0.10) was detected for BW (Table 7). On day 21, HOSBO steers tended (*P* = 0.10) to have greater BW than steers in the other treatments. Additionally, on day 56, HOSBO steers tended (*P* = 0.10) to have greater BW than CSBO steers, which in turn tended (*P* = 0.10) to have greater BW than Control steers. Furthermore, a similar pattern was observed for ADG. From day 0 to 21, HOSBO steers tended (*P* = 0.10) to have greater ADG than steers receiving other treatments. Although no treatment effects were detected from day 21 to 56 (*P* = 0.39), HOSBO steers tended (*P* = 0.10) to have greater ADG than Control steers over the entire period (day 0 to 56), whereas CSBO steers did not differ from the other groups.

**Table 7:** Body weight (BW) and average daily gain (ADG) of beef steers supplemented with no-oil (Control; *n* = 24), conventional soybean oil (CSBO; *n* = 24), or high-oleic soybean oil (HOSBO; *n* = 24) during the 56-day preconditioning period^1^.

Items	Treatments			SEM	*P*-value	
	Control	CSBO	HOSBO		Treatment	Treatment× day
**BW, kg^2^**					0.05	0.10
**d 0**	248	247	248	1.50		
**d 21**	256^b^	256^b^	260^a^	1.50		
**d 56**	271^c^	274^b^	278^a^	1.55		
**ADG, kg/d**						
**d 0–21**	0.38^b^	0.43^b^	0.57^a^	0.06	0.10	–
**d 21–56**	0.43	0.51	0.51	0.06	0.39	–
**d 0–56**	0.41^b^	0.48^ab^	0.54^a^	0.04	0.10	–

1 Hay was offered ad libitum, and soybean hulls were fed at 0.8% of body weight (BW). The Control group received no oil, whereas the CSBO and HOSBO groups had oil top-dressed onto soybean hulls at a rate of 0.06% of BW per steer per day. The day 0 values were included as a covariate in the statistical model.

2 Day 0 values were included as a covariate in the statistical model (*P* ≤ 0.01).

a,b,c Within a row, without a common superscript differ (*P* ≤ 0.05) or tend to differ (*P* ≤ 0.10).

## Discussion

Previous research demonstrated that preconditioned steers tend to gain more weight and exhibit better health during the receiving period, and that the nutritional management used during preconditioning is one of the primary factors influencing the effectiveness of this practice (Galyean et al. 2022). Because CSBO and HOSBO differ in their FA profiles, they are expected to exert distinct effects on immune function and performance, and to our knowledge, no previous studies have compared these oils in this specific animal category.

The current experiment has two important limitations that must be explicitly acknowledged. The first limitation is the absence of direct measurements of DM intake (DMI). The use of round bales in hay rings prevented accurate quantification of hay disappearance and, consequently, of total diet composition consumed by steers in each pasture. However, using NASEM (2016) equations to estimate hay intake, knowing the quantity of soybean hulls and oils offered and the analyzed composition of the ingredients, we estimate that Control steers likely consumed a total diet containing approximately 8.80% CP, 2.20% EE, and 57% TDN, whereas CSBO and HOSBO steers likely consumed diets containing approximately 8.60% CP, 5.30% EE, and 61% TDN. These values, however, remain estimates and cannot replace direct DMI measurements. The second limitation is the low CP concentration (<9%) in the basal diet, which is below the requirements of newly weaned calves and has constrained growth. Even so, this diet was selected because it reflects what producers commonly use during preconditioning in Louisiana.

In our experiment, we observed that supplementing soybean oil at 0.06% of BW per steer per day had minimal effects on rumen microbiology but markedly altered plasma FA profiles, with CSBO increasing linoleic acid and HOSBO increasing oleic acid concentrations. These alterations were paralleled by distinct immune responses, as CSBO enhanced the antibody response to BVDV, whereas HOSBO enhanced the response to BRSV on day 21. In addition, HOSBO was more effective than CSBO in supporting the growth of recently weaned Brangus steers during the initial 21 days on feed. This early growth advantage likely reflects the period during which steers experienced the greatest physiological stress and immune activation following weaning and vaccination (Lynch et al. 2019; Galyean et al. 2022). During the first three weeks, calves face heightened metabolic and inflammatory demands, and the greater availability of oleic acid from HOSBO may have supported more efficient immune regulation and cellular metabolism, allowing more nutrients to be partitioned toward growth (Santa-María et al. 2023). As the preconditioning period progressed and physiological stress subsided, the relative advantage of HOSBO diminished.

Feeding PUFA-rich oils such as CSBO is known to result in greater ruminal biohydrogenation and more pronounced alterations in rumen microbiology compared with high-MUFA oils such as HOSBO (Jenkins 1993; Jenkins et al. 2008; Jenkins and Harvatine 2014; Lock et al. 2025). However, in our experiment, the HOSBO did not affect rumen bacterial communities, whereas CSBO reduced genus-level alpha diversity by lowering richness and tending to decrease the Shannon index. These patterns suggest that linoleic acid–rich oil may selectively suppress less abundant taxa without altering dominant bacterial groups. This interpretation is supported by the lack of treatment effects on the Simpson index, indicating stable community dominance across treatments. Greater inclusion of CSBO or linseed oil is known to increase proteolytic and decrease cellulolytic bacteria and protozoa in the rumen fluid (Yang et al. 2009). However, the minimal microbial shifts observed in our study likely reflect the relatively safe oil feeding rate used. Steers consuming oil had an estimated EE concentration of approximately 5.26% in the total diet; below the 6% maximum threshold recommended (NASEM 2016).

Supplementation with CSBO and HOSBO markedly altered the plasma FA profile of recently weaned steers, and the direction of these changes closely reflected the distinct FA composition of each oil source. As expected, CSBO, rich in linoleic acid, resulted in substantial increases in plasma linoleic acid on days 21 and 56, whereas HOSBO, rich in oleic acid, led to pronounced increases in plasma oleic acid. These responses are consistent with classical ruminal lipid metabolism, in which dietary PUFA and MUFA undergo rapid lipolysis followed by extensive biohydrogenation by rumen microorganisms (Lock et al. 2025). However, biohydrogenation is more extensive for PUFA than for MUFA, allowing a greater proportion of MUFA to escape the rumen and be absorbed post-ruminally (Jenkins 1993; Jenkins et al. 2008; Jenkins and Harvatine 2014). In an experiment with beef steers, HOSBO supplementation led to a greater concentration of oleic acid in the abomasum than CSBO supplementation, supporting this explanation (Shirley 2019).

The greater plasma stearic acid concentration in HOSBO-fed steers on day 21 was unexpected but likely reflects more efficient biohydrogenation of oleic acid early in the supplementation period. In contrast, the subsequent increase in stearic acid observed in CSBO-fed steers by day 56 may be explained by microbial adaptation and by the greater cumulative supply of linoleic acid available for biohydrogenation over time. Linoleic acid is more extensively biohydrogenated in the rumen (approximately 80 to 95%) compared with oleic acid (approximately 60 to 80% [Lock et al. 2025]). Therefore, as steers adapted to the diet and continued to receive a greater supply of linoleic acid from CSBO, greater production of stearic acid would be expected. Interestingly, HOSBO-fed steers exhibited greatest plasma α-linolenic acid on day 21, despite HOSBO being low in ω-3 PUFA. This may reflect reduced microbial utilization or altered ruminal metabolism of endogenous dietary ω-3 FA when oleic acid is supplemented. No other experiments evaluating HOSBO on plasma FA concentrations in cattle were found in the literature for comparison, and further research is warranted.

Plasma cortisol concentrations were transiently elevated in CSBO steers on day 21, compared with the Control group. This response coincided with increased ω-6 PUFA availability and may reflect interactions between FA mediated inflammatory signaling and hypothalamic–pituitary–adrenal axis activation (Poli et al. 2023). In addition, CSBO steers tended to have greater serum AST concentrations on day 56, which could indicate mild hepatic stress or increased metabolic load, although values remained within normal physiological ranges (Kaneko 2008).

One of the most notable findings of this study was the divergent response to vaccination between oil sources. Steers supplemented with CSBO exhibited greater antibody titers against BVDV-1 and BVDV-2, whereas HOSBO selectively enhanced the BRSV antibody response at day 21 post-vaccination. It is important to note that the overall response to the BRSV vaccine was low across all treatments, with seroconversion rates below 51%. No issues related to vaccine handling, storage, or administration were observed, and the reduced response to BRSV was specific to BRSV, as the same animals mounted adequate responses to the other viral antigens included in the vaccine. This pattern suggests that factors intrinsic to the BRSV antigen or to the biological characteristics of this virus may have contributed to the generally poor immunological response. Furthermore, to our knowledge, no previous studies have directly compared CSBO and HOSBO on vaccine-induced immune responses in livestock. However, literature demonstrates that different classes of FA exert distinct effects on immune cell function, inflammatory signaling, and antibody production, suggesting that plasma FA profile may differentially modulate immune responses depending on the antigenic or viral challenge (Calder 2008; Radzikowska et al. 2019).

The CSBO increased circulating linoleic acid, the main dietary precursor of arachidonic acid, which is highly enriched in immune cell membranes and serves as a substrate for eicosanoid synthesis (Calder 2008; Radzikowska et al. 2019). Arachidonic acid-derived eicosanoids play a central role in lymphocyte activation, antigen presentation, and antibody production, potentially amplifying humoral immune responses following vaccination (Calder 2008; Radzikowska et al. 2019). That enrichment of immune cells with ω-6 FA enhances eicosanoid production capacity and can increase the magnitude of immune responses under stimulatory conditions (Calder 2008), providing a mechanistic basis for the greater BVDV-1 and BVDV-2 titers observed in CSBO-supplemented steers.

In contrast, HOSBO supplementation increased oleic acid availability, a MUFA associated with more regulated immune activation and reduced susceptibility to lipid peroxidation (Radzikowska et al. 2019). Because oleic acid is less prone to oxidative damage than PUFA, its incorporation into immune cells likely reduced oxidative stress during activation (Yang et al. 2022; Santa-María et al. 2023), helping preserve membrane integrity, receptor signaling, and lymphocyte function; key elements for coordinated innate and adaptive immunity (Calder 2008; Radzikowska et al. 2019). In parallel, oleic acid activates anti-inflammatory and antioxidant pathways, while suppressing proinflammatory cytokine production (Santa-María et al. 2023), thereby promoting a more regulated immune environment that supports efficient adaptive immune responses. Collectively, these mechanisms help explain the greater serological response to BRSV observed in calves receiving HOSBO.

The distinct FA profiles provided by the two oil sources tested in our experiment likely contributed to the divergent vaccine responses observed. These findings highlight that immune responses to vaccination may vary depending on the predominant FA in the diet. Therefore, further research directly comparing how specific FA classes modulate immunity to different viral antigens is needed before recommending one oil source over another based on the predominant pathogens present within a given production system.

Both oil supplements tended to increase BW and ADG, with a greater response in HOSBO steers than in CSBO steers, particularly during the early post-weaning phase. Studies evaluating HOSBO in beef cattle diets are scarce, with only one study reported in the literature (Shirley 2019). In that experiment, supplementing with 3% HOSBO, rather than CSBO, did not affect the intake or growth performance of confined finishing steers (Shirley 2019). Likewise, another study reported that feeding 5% of the diet as either high-linoleic or high-oleic safflower oil did not alter the growth performance of finishing steers (Hristov et al. 2005). Comparing our results with previous studies indicates that the growth response to HOSBO is context dependent, likely reflecting differences in basal diet composition, oil inclusion level, and animal category, as previously discussed.

The tendency toward greater ADG observed in HOSBO-supplemented steers vs CSBO-supplemented steers in our experiment, particularly during the early post-weaning phase (initial 21 days), could be related to differences in DMI, which were not measured. Additionally, this response appears to be related to differences in systemic FA profiles and metabolic stress (Santa-María et al. 2023). High-oleic soybean oil increased circulating oleic acid and maintained a lower ω-6/ω-3 PUFA ratio compared with CSBO, which increased linoleic acid availability. In contrast to CSBO, HOSBO supplementation was not associated with a transient increase in cortisol or elevated liver enzyme activity, suggesting a lower metabolic and inflammatory load (Santa-María et al. 2023). Reduced stress and inflammatory demands during the post-weaning period (fundamental conditions for proper gain [Galyean et al. 2022]) have allowed greater nutrient partitioning toward growth, thereby resulting in a tendency to improve ADG in HOSBO-supplemented steers.

## Conclusions

Supplementing soybean oil at the evaluated inclusion level had minimal effect on rumen microbiology but altered circulating FA profiles in recently weaned Brangus steers during a 56-day preconditioning phase. Conventional soybean oil increased plasma linoleic acid concentrations and enhanced antibody responses to BVDV, whereas HOSBO increased plasma oleic acid concentrations and improved antibody responses to BRSV on day 21. Steers receiving CSBO tended to have greater serum AST concentrations than those receiving HOSBO. In contrast, steers supplemented with HOSBO tended to have greater BW and ADG than those receiving CSBO, particularly during the early preconditioning phase.

## List of abbreviations

ADF: Acid detergent fiber
ADG: Average daily gain
ALT: Alanine aminotransferase
AST: Aspartate aminotransferase
BRSV: Bovine respiratory syncytial virus
BVDV: Bovine viral diarrhea virus
BVDV-1: Bovine viral diarrhea virus type 1
BVDV-2: Bovine viral diarrhea virus type 2
BW: Body weight
CSBO: Conventional soybean oil
CP: Crude protein
DM: Dry matter
DMI: Dry matter intake
EE: Ether extract
FA: Fatty acids
GGT: Gamma-glutamyl transferase
HOSBO: High-oleic soybean oil
IBR: Infectious bovine rhinotracheitis
IGF-1: Insulin-like growth factor 1
NDF: Neutral detergent fiber
NEg: Net energy for gain
NEm: Net energy for maintenance
MUFA: Monounsaturated fatty acids
PI_3_: Parainfluenza-3 virus
PUFA: Polyunsaturated fatty acids
SFA: Saturate fatty acids
TDN: Total digestible nutrients
